# Costs and renumeration of osteomyelitis treatment involving free flaps: implications of return to theatre

**DOI:** 10.7150/jbji.22186

**Published:** 2018-02-05

**Authors:** Rebecca Shirley, Janka Fazekas, Martin McNally, Alex Ramsden

**Affiliations:** Nuffield Orthopaedic Centre, Windmill Road, Headington, Oxford, OX3 7HE

**Keywords:** Osteomyelitis, Free flap failure, Return to theatre, Cost analysis, Healthcare Resource Group

## Abstract

**Aim**: This study aimed to define the costs of surgical management of chronic osteomyelitis where free tissue transfer was required in addition to debridement of bone, particularly the increased costs incurred by a return to theatre. We hypothesised that there would be a significantly greater cost when patients required re-exploration for vascular compromise.

**Method**: We retrospectively analysed the costs of a consecutive series of sixty patient episodes treated at the Bone Infection Unit in Oxford from 2012 to 2015. Treatment involved excision of osteomyelitis with free tissue transfer for immediate soft tissue cover. We compared the costs of uncomplicated cases with those who returned to theatre and determined the profit / loss for the hospital from renumeration through the UK National Health Service Tariff Structure.

**Results**: Hospital income according to UK HRG tariff was compared to the actual cost of treatment and these 60 cases were significantly underfunded overall (P < 0.005). In just 1 case, the cost to the hospital was completely covered by tariff.

Six patients (10%) returned to theatre for urgent flap re-exploration with five flaps salvaged and one failed, requiring another free flap reconstruction (1.7%). These six patient episodes had a significantly higher mean cost compared to the uncomplicated cases. The average financial loss to the hospital for patients who did return to theatre was £19401 (range £8103 to £48380) and in those who did not was £9600 (range - £600 to £23717). The case requiring further free tissue transfer cost a total of £74158, £48380 more than the hospital was paid: the most extreme discrepancy. The overall loss for this group of 60 patients was £610 090.

**Conclusions**: Surgery for chronic osteomyelitis is multidisciplinary, complex and therefore expensive with a significant risk of complications. However, this study demonstrates that the hospital currently makes a financial loss on almost all patients but especially if flap complications occur. This study has implications for the long term viability of specialist units treating this important disease.

## Introduction

“Payment by results” is the national funding system within the National Health Service (NHS), introduced in 2003^1^. Primary care trusts pay hospitals to deliver services; hospitals receive money for each episode of care, rather than receiving a set amount of money under block contracts. The system necessitates accurate collection of data or clinical coding as this is used as evidence for payment of the work undertaken and is also useful for audit purposes. Coding is carried out by professionally trained coders, who rely on the accuracy of clinicians notes for diagnoses and procedures.

Each patient encounter is allocated a Healthcare Resource Group (HRG) code based on the detail of the encounter. Each HRG is associated with a national tariff and each HRG code is based on a complex algorithm.

Diagnoses are coded based on international statistical classification of diseases and other health problems, 10th edition (ICD 10) set out by the World Health Organisation. Procedures are coded using the Office of Population, censuses and surveys Classification of Surgical operations and procedures (OPCS-4).

For patients having multiple procedures, each procedure is ranked and the highest will be used to generate the HRG code. The ranking was set by “expert working groups”, which included physicians.

For each patient encounter, diagnoses and procedure codes are entered into a computer system to generate an HRG code. Each HRG has a “trim point” which is the number of days expected in hospital for the treatment. If a patient's stay is longer than the trim point, generally 42 days for these cases, an additional £260 a day is allocated. This is generally inadequate when compared to the actual cost of approximately £400 per day on the bone infection unit. Some devices and implants can be charged as an additional expense outside tariff, such as the use of customized implants or circular external fixators.

Tariffs (HRGs) are based on average costs; some cohorts of activity will be more expensive and this is the case for patients undergoing two simultaneous complex procedures. Factors such as co-morbidities and complexity will affect an HRG, so the same procedure carried out on patients with none or varying co-morbidities/complexities will determine the outcome of the HRG.

The provision of bone infection care is complex and patients are referred to our unit from all over the UK, often having undergone numerous operations over many years. We hypothesized that the fixed priced payment system (based on average costs), would not account for the complexity and high cost of this challenging condition. The shortfall in payment has already been clearly demonstrated for acute open tibial fractures requiring free tissue for cover^2^ This unit integrates three clinical teams (orthopaedic and plastic surgery with bone infection physicians) with plastic surgeons involved in approximately 30% of the cases referred. Due to the nature of complex revisional surgery, we anticipate a higher rate of complications than acute, non infected cases.

There is some provision for the complexity of the cases. HRGs ending in “34” attract 28% Trauma and Orthopaedic top up payment. This specialist top up is applicable to a number of episodes in this study and only those Trusts designated by the DoH as specialist are able to obtain the top up payments. A certain procedure can generate a different tariff depending on varying co-morbidities and patient factors. Details of a patient's diagnosis and management go through a clinical coding process.

The aim of this study was to assess the cost of treatment for patients undergoing osteomyelitis excision with free flap reconstruction and compare it to the renumeration. The tariffs are complex and variable and the focus of this article is to explain them in full. The tariffs vary within the group, but for example a very common tariff applied in this series is £3650 and the expected hospital stay is 42 days. If the hospital stay exceeds this, an additional daily rate is added to the tariff.

We were particularly interested in the small group of patients requiring additional unplanned surgery during the episode of care. We anticipated the complication rate to be high given the often longstanding chronic inflammation and multiple previous procedures, making microsurgery more challenging than in acute circumstances. Although microvascular surgery and free tissue transfer is well established surgery is complex and it is accepted complications including the need for urgent revascularization in inevitable in a small proportion of patients^3^. We hypothesised further emergency surgery would significantly add to the total cost of treatment and not be adequately accounted for in the renumeration.

The coding system is not affected by the success or failure of a procedure, so many patients who have undergone procedures previously will have already cost the NHS significant monetary and clinical resources but still require definitive treatment. This means it is possible for a hospital to make a profit on simpler unsuccessful surgery, while losing money on more complex, definitive and curative surgery.

## Method

We retrospectively reviewed the costs and outcomes of 60 consecutive patients who have undergone excision of osteomyelitis and free flap reconstruction at The Nuffield Orthopaedic Centre (NOC), a tertiary referral centre for osteomyelitis. The operations took place between 2012 and 2015; the reason for starting in 2012 was that before this the data collection from the costing department was incomplete.

In this series the majority of patients underwent lower limb surgery under epidural using a gracilis muscle flap for soft tissue cover. This is safer and more cost effective than general anaesthesia, avoiding the use of inhalational gases. The unit has a long established routine use of invasive doppler monitoring, which allows early detecting of vascular problems, particularly in muscle flaps where clinical flap monitoring is more difficult^2^. It maximises the chance of flap salvage. A recent meta analysis suggested invasive doppler increased the chance of flap salvage but did result in a higher rate of unnecessary to return to theatre^4^.

Our free flap database was used to identify 60 consecutive cases of free tissue transfer used to treat osteomyelitis at the NOC. The unit does not have paediatric beds, therefore no children were included in this study. There were no other exclusion criteria and all patients operated in the time frame were included. The procedures were performed by one of four lead surgeons. The database also contains demographics, flap choices, operation time and information about patients requiring return to theatre.

We gained information about cost of treatment from the hospital finance department. This was derived from a Patient-Level Information and Costing System (PLICS) which included all costs; costs of theatre time and ward costs, allowing for staff salaries and repayments for private finance initiatives. Other costs including radiology, pathology, medication and expendable materials are also included.

We were able to obtain the total costs for all patients from the clinical coding department. We also referred to the computerised medical records to glean further information and cross check the accuracy of the records held by the coding department and the free flap database.

We ascertained the cost to the hospital of all sixty episodes: the total in-patient stay with all additional costs included and compared this total with the re-numeration for each patient.

A paired T test was used to compare the cost of the procedures and the re-numeration generated. We also compared the cost of the patients requiring further surgery, with those who did not.

## Results

The study group consisted of 60 consecutive patients all treated for chronic osteomyelitis necessitating simultaneous surgical debridement and free tissue transfer. Overall there were 16 females and 46 males. Age ranged from 19 to 82 (mean age 49)

The free flaps included 47 gracilis flaps, 7 latissimus dorsi (LD), 5 fibula flaps and 1 anterior lateral thigh (ALT) flap.

15 patients required external fixation: for stabilization in fourteen cases and bone transport in 1 case. 5/15 had monolateral fixators and the remainder had circular fixators. Three of the cases requiring external fixation also required return to theatre for re-exploration of the flap.

### Cases requiring urgent further surgery

Six patients (10%) returned to theatre for urgent flap re-exploration with five flaps salvaged (8.3%) and one failed, requiring another free flap reconstruction (1.7%). Four free flaps were salvaged with one further operation in each case, one was salvaged with two further operations and one was not salvaged, requiring three further operations, including another free flap. None of these six patients have had recurrence of osteomyelitis and consequently successful outcomes at the end of 2015.

The figure shows the mean cost for the uncomplicated cases in the first column, comparing it to those requiring further surgery. It is evident that unplanned surgery increases the overall cost of the encounter and that each operation increases the overall cost more.

The mean cost to the hospital for the 54 uncomplicated cases was £16,678, with a standard deviation of 5629.05. The mean cost for the 5 cases in which an early return to theatre led to flap salvage was £20,845 with a standard deviation of 10474.45.

The one patient who had complete failure of the flap, followed by three additional surgical procedures required an in patient stay of ninety days. The first operation was attempted re-vascularisation of the flap, the next procedure: debridement of the flap and the last, a further free flap. The total cost for the episode was £74158.This was nearly four times the amount of money the hospital received for that episode and more than double the cost of any of the other 59 patient episodes. Both the hospital stay and the cost of operating time contributed significantly to the increased cost. Although this patient also attracted the greatest total funding as a result of the complication and hence longer hospital stay, it was not nearly sufficient to cover the actual excess cost. A table shows a break down of the costs for this patient.

It is important to state that all six of these patients went on to have good outcomes and as of July 2017, none of these cases have had recurrence of osteomyelitis. Three of the cases required removal of metal work and one required a revision of a custom made endoprosthesis. These late procedures were all anticipated and other than that, there were no further operations after discharge in the six patients.

### One profit making case

There was one patient whose treatment costs were covered by the HRG code. The patient was young with no co-morbidities. His treatment was uncomplicated. He had a free fibula flap with a monotube external fixator and was an inpatient for 8 days.

### Breakdown of costs

The total costs of treatment are mainly comprised of ward costs and theatre costs. There are additional costs for radiology, pathology, management and administration and pharmacy. Pharmacy costs can become significant when expensive antibiotics are indicated. In fact a separate audit within the department showed £156,000 spent on antibiotics to 64 patients, an average of £2300 per patient.

### High cost cases

We also looked at patients whose hospital stay exceeded fifty days, this was the case for two patients who did not have surgical complications. One patient was living abroad and had a two stage procedure and therefore had to stay as an inpatient for the removal of an infected IM nail and subsequent reconstruction. This was the only patient in the group who did not require additional surgery but that still cost more than £30,000.

The other patient had Behcet's disease, was immunosuppressed and had significant pain post operatively which required a prolonged hospital stay before being ready for discharge.

## Discussion

The recent Getting it right first time (GIRFT) report from the British Orthopaedic Association revealed that deep wound infection in total knee and hip surgery currently runs as high as 5% for a number of units and costs approximately £100,000 per patient^5^ (based on comparative cost analysis of managing deep wound infection across a number of providers - cost drivers including re-operation, extended length of stay, high cost long term antibiotics and new high cost replacement prostheses). Treatment of these infections adds an extra £1,000 for each arthroplasty procedure to cover the costs of readmission, re-operation and medication for infected patients, as well as costs in the community = £1.5 billion over 5 years. This figure would be added to by complications of all other orthopaedic procedures, however it is not possible to quantify this. When osteomyelitis occurs after infection of an open tibial fracture it is a devastating condition and accounts for long absenteeism. It increases the cost of open tibial fracture by 60%, as well as doubling the length of hospital stay^6^.

Our population group all had established osteomyelitis with associated soft tissue defect, frequently having had unsuccessful previous operations over many years. At present the value of successful treatment is not reflected in the funding system.

Our study demonstrates a gross difference between the actual cost of treatment for this group of tertiary referral patients and the re-numeration that has been allocated according to HRG coding. This is despite our Unit providing a combined service between specialties, giving single-stage treatment where possible and limiting in-patient stays. We have developed a home intravenous therapy service, to reduce cost and now switch patients to very early oral therapy, avoiding expensive intravenous antibiotics. It would be possible to increase the remuneration to the hospital by having multiple episodes of care (separate infection excision, soft-tissue cover and bone reconstruction); each one generating a separate tariff and new income for the hospital. This is a common situation around the world, but is clearly not in the patients' best interests.

The study also shows that when compromised flaps are salvaged quickly there is not a significantly higher price to pay, although the necessary theatre time is expensive (£1000 an hour). The 5 patients who had early flap salvage did not have a significantly greater mean cost in the study group. On the other hand, in the one case where the free flap was not salvaged there was an extremely high cost. The patient had three further operations, including the second free flap and the associated 90 day stay. Although the additional hospital stay did result in an extra nightly payment this was much less than the actual cost and this was by far the most underfunded case.

The accuracy of coding is another concern outside the remit of this paper. A recent study highlighted important coding errors that led to a significant loss of revenue in an ENT department in York^7^. There is also the problem, that there are no codes available for combined surgical procedures which include microvascular tissue transfer and complex bone reconstruction for osteomyelitis.

## Conclusions

There is a considerable disparity between the cost of effectively managing osteomyelitis and the remuneration according to the HRG code. Accurate cost analysis of these cases shows that the current tariffs are not nearly sufficient to cover the cost of treatment, even when there are no complications. This resulted in a total shortfall of £610,090 for all 60 cases This information is evidence for negotiation of proper renumeration.

The results of our cost analysis demonstrate that chronic osteomyelitis patients require intensive resource and service input due to high rates of complication, prolonged in-hospital stays, long complex procedures and involvement of senior colleagues across different teams. Although there is some provision for the complexity of the cases, reflected in the HRG coding, even in the uncomplicated cases it is insufficient to cover the actual cost of this work. The study demonstrates that the cost of osteomyelitis treatment is underfunded.

Our study shows that flap loss, which occurred in 1 of 60 cases was extremely expensive and not provided for in the funding system. It demonstrates the importance of flap monitoring, as in the five cases of successful salvage of compromised flaps, the additional cost was proportionately much less than the potential cost of losing a flap and hospital stay was not particularly longer where a flap was salvaged.

The disparity will become an increasing problem for the NHS with increasing privatization. There is a clear incentive for non-specialist Units to perform simple treatments without adequate soft tissue or bony reconstruction, even if they have a low chance of success. The tariff system will cover the cost of this ineffective treatment and eventually, the patient can be referred to a more specialist Unit which will need to bear the loss. This will inevitably mean that these complex poorly re-numerated cases will form an increasing burden to the NHS.

## Figures and Tables

**Figure 1:**
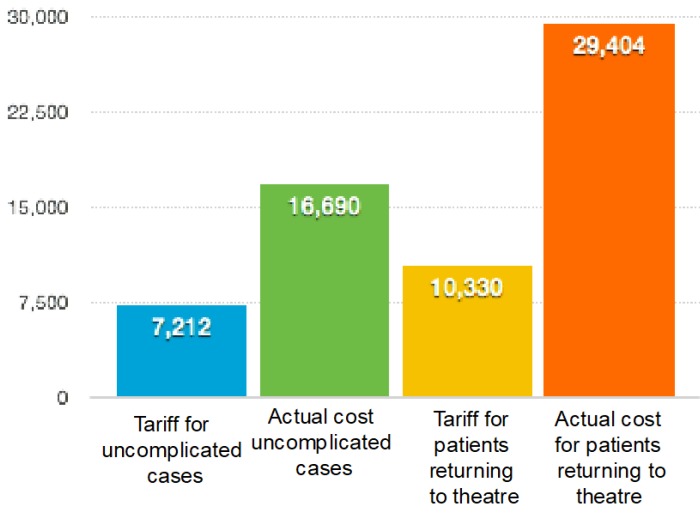
Graph to show variation of mean costs in pounds paid to the trust and the actual cost of the treatment in uncomplicated cases (columns A and B) and those requiring further surgery (columns C and D)

**Figure 2:**
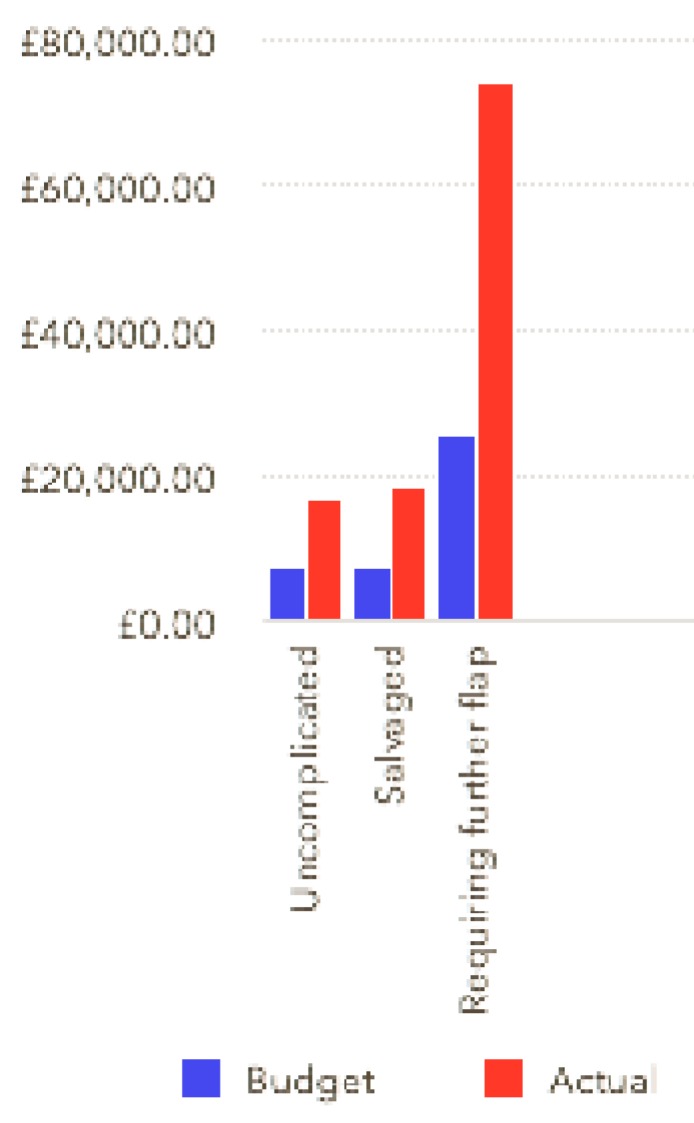
showing variation in budget: the tariff received by the trust and expenditure. The tariff received is shown in blue and the actual cost in red. The first two columns are mean costs for the 56 uncomplicated cases, the second two are mean values for the five salvaged cases and the last columns are the figures for the one cases requiring a second free flap.

**Figure 3:**
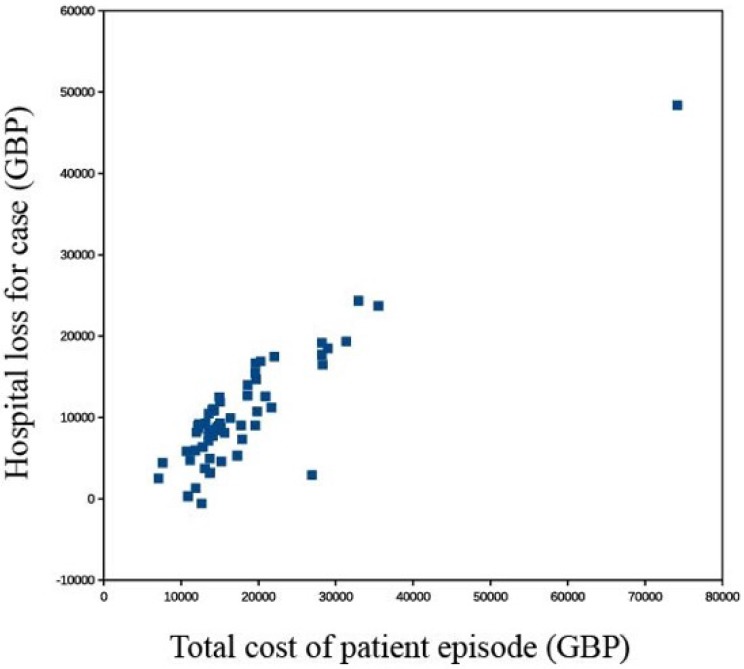
Cost of patient episode compared to total loss to the hospital. Compared to total: The one patient who had a failed free flap and required another free flap is a gross outlier, it was the most expensive episode, just over £74 000 and had the highest deficit, more than £48 000. This scattergraph shows there is a correlation between the cost of an episode and the short fall in funding: more expensive episodes are more underfunded. One of the 60 episodes made a profit for the hospital.

**Table 1:** summarising 6 patients who required urgent re-operation

	Sex	Age	Site of osteomyelitis	Flap	Outcome	Length of stay after surgery (days)	Number of re-operations during admission	Re-numeration (£)	Actual cost of treatment (£)
1	M	72	Upper limb	Gracilis	Salvaged	20	1	8103	15648
2	M	50	Lower limb	Gracilis	Salvaged	13	1	5829	10721
3	M	49	Lower Limb	Gracilis	Salvaged	16	1	10439	13573
4	F	49	Lower Limb	Gracilis	Salvaged	28	1	24332	32925
5	F	36	Upper Limb	Lat Dorsi	Salvaged	38	2	19324	31359
6	M	63	Lower Limb	Gracilis	Failed	90	3	48380	74158
